# Modified use of the proximal humeral internal locking system (PHILOS) plate for distal femoral nonunions

**DOI:** 10.1007/s00590-022-03203-4

**Published:** 2022-01-21

**Authors:** Josje Poelmann, Peter Kloen

**Affiliations:** Department of Orthopedic Surgery, Amsterdam University Medical Center, Meiberdreef 9, Amsterdam, 1105AZ The Netherlands

**Keywords:** Distal femoral fracture, Nonunion, Dual Plating, Philos

## Abstract

**Purpose:**

Nonunion is a common complication after a distal femoral fracture (DFF). Standard treatment consists of revision plating and/or bone grafting. Single lateral plating for a distal femoral nonunion can be insufficient in case of a persistent medial gap and compromised bone stock. Alternatively, dual plating can be used to treat a distal femoral nonunion, but to date there is no Gold standard. The aim of our study was to report our results after use of a minimally invasively placed proximal humeral internal locking system (Philos) plate as a medial buttress in the treatment of a distal femoral nonunion.

**Methods:**

Fifteen adult patients with a distal femoral nonunion were prospectively entered in a trauma database and retrospectively assessed. All patients underwent a similar operation, which included removal of failed hardware, nonunion debridement, fixation with a lateral plate, and a medial Philos plate combined with bone grafting. Data collected included union rate, time to union, complications and functional outcome.

**Results:**

In twelve out of fifteen patients (80%), the fracture united after our index operation. Median time to union was 4.8 months (range 1.6–15). Three patients (20%) needed additional bone grafting surgery. One patient underwent a Judet quadricepsplasty.

**Conclusion:**

This study suggests that the Philos plate is a safe and effective adjunct as a medial buttress plate for distal femoral nonunions.

## Introduction

Distal femoral fractures (DFF) account for about 5% of all femoral fractures. The incidence will continue to grow as the population ages [1, 2]. These fractures are most often treated using a (minimally invasive) locking compression plate (LCP) or retrograde intramedullary nail (RIMN) [3, 4]. DFFs are often intra-articular fractures with metaphyseal comminution, which makes achieving adequate reduction challenging [5]. In addition, the short distal femur fragment, the proximity of the knee joint, and the poor bone quality predispose to complications after fixation [6, 7].

Numerous studies have reported on nonunion following a DFF, with rates varying widely (0–31.8%) [3]. Nowadays, a pragmatic definition is used: “a nonunion is a fracture that will not heal without further surgical intervention”. This definition shortens the classic time of 9 months to intervention significantly. However, this definition leaves an interpretation gap between the treating surgeons.

Nonunions of the distal femur often present with loss of vital bone stock, osteopenia and stiffness. A persistent medial cortical gap leads to inadequate support with progressive varus malalignment resulting in collapse and hardware failure [8–10]. Literature suggests salvage with addition of a medial plate if single lateral plating is insufficient, but there is no consensus on which plate type to use [10–13].

We have used the proximal humeral internal locking system (Philos) plate for this location. This plate was originally designed for the proximal part of the humerus, but its flexibility and shape allow for application in other parts of the human body [14, 15]. To the best of our knowledge, there are no studies on the use of dual-plate fixation using the Philos plate for distal femoral nonunions (DFN).

The aim of our study was to investigate the applicability and outcome of the Philos plate in dual plating of distal femoral nonunions.

## Methods

### Patient cohort

For our retrospective cohort study, we searched the database of senior author’s logbook for patients treated for a DFN. Fifteen patients met the following inclusion criteria: (1) persistent nonunion after initial DFF treatment, (2) surgical treatment consisting of dual plating with a Philos plate on the medial side of the distal femur, (3) minimum age of 18 years old at the time of surgery. Exclusion criteria were: (1) no informed consent, (2) lack of previous DFF treatment history and/or incomplete files, (3) pathologic fractures. We obtained IRB-approval.

The first surgical intervention to procure healing was considered the initial DFF treatment and was often (13/15) performed in another institution. The surgical intervention using the Philos plate was considered the index procedure. Two patients in our cohort received all interventions and follow-up appointments in another medical facility, but were operated on by the senior author. As we had sufficient follow-up data, they were included in our study. Patient demographics, treatment history and fracture characteristics were documented. The nonunion severity score (NUSS) was calculated [16].

### Surgical technique

In eight patients, we started harvesting posterior iliac crest bone graft (ICBG) because the anterior ICBG had already been used previously, or because we anticipated the need for a large (up to 6–7 cm) tricortical graft. No antibiotics were administered before five deep cultures from the nonunion site were taken. All failed hardware was removed; lateral plates remained in situ if they were stable. The prosthetic implant in four patients was checked intra-operatively. None were loose. Adhesions between quadriceps and femur were removed. Non-viable fragments were aggressively debrided using a scalpel, rongeur or electrocautery. The nonunion was opened and the bone marrow canal was drilled anterograde and retrograde until blood was seen to egress. An area of 2.5 cm on the visible anterolateral and posterolateral surface was petalled with a sharp osteotome to increase bleeding bone surface. Large defects were filled with either tricortical ICBG, autologous grafts or allografts from the bone bank.

**Fig. 1:**
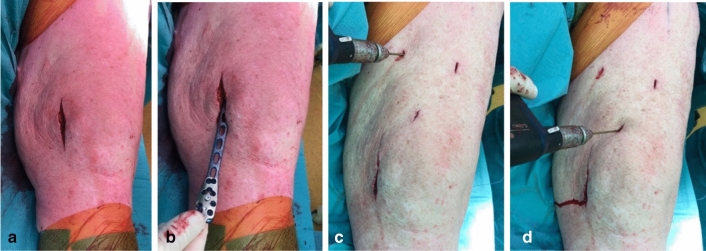
*Application of the Philos plate on the distal femur*. Medial incision on the distal femur (**a**). The Philos plate which is inserted along the medial border using a small single incision (**b**) Percutaneous minimal invasive attachment of the plate to the femur (**c**, **d**)

For lateral fixation, we used different plate types being a variable angle (VA) distal femur lateral condylar plate (VA-LCP, DePuy Synthes, Amersfoort, the Netherlands), a 95-degree condylar blade plate (DePuy Synthes), a less invasive stabilization system (LISS, DePuy Synthes) plate or an AxSOS locking plate (Stryker, Amsterdam, the Netherlands). After correction of alignment, the AO-tensioner device was used to compress the nonunion while maintaining alignment. The plate was then fixated with hybrid fixation. Using a minimal invasive technique the Philos plate was inserted from distal to proximal along the medial side of the distal femur (Fig. 1). Proximally the plate was percutaneously fixed using cortical screws, distally using locking screws.

### Post-operative management and follow-up

On post-operative day one, the patient started toe-touch weight bearing. Continuous passive motion (CPM) machine was used for three patients because they had undergone extensive adhesiolysis. Antibiotic use was continued until all cultures were proven negative. If two or more of five cultures resulted positive a personalized plan to treat infection was obtained. Toe-touch weight baring was allowed for 6 weeks after operation, approval for further increase in weightbearing was given if patients were in good clinical condition and/or radiographic imaging showed sufficient strength of the bone. Follow up was performed at 6 weeks, 3 months, 6 months and 1 year after surgery. In addition, we used the Knee Injury and Osteoarthritis Outcome Score (KOOS) [17] as a patient derived outcome score.

## Results

We identified 15 patients (9 females) with a median age of 68 (range 19–81) who underwent surgery for a DFN using the Philos plate as a medial buttress between November 2013 and October 2019. Fall from standing height was the most common low energy trauma (LET) cause (*n* = 5). A motor vehicle accident (MVA) was the fracture cause for all patients involved in a high-energy trauma (HET) (*n* = 6). In one patient, mechanism of injury (MOI) was not specified. Fractures were classified using the AO/OTA classification system. Four patients sustained a DFF above total knee prosthesis. Fractures were classified as open (4/15) or closed (11/15) according to the Gustilo-Anderson Classification system. Three patients presented with a broken plate. For specifications and additional demographics, see Table 1.

**Table 1:** Patient and fracture characteristics

Patient	Age	Sex	ASA	Side	MOI	AO/OTA	Prosthesis	Open/Closed*
1	19	M	1	Left	HET	33C3	–	Open, grade II
2	30	M	1	Left	HET	33C3	–	Closed
3	40	M	2	Left	HET	33C3	–	Open, grade IIIA
4	49	M	2	Right	HET	33C3	–	Open, grade IIIA
5	55	F	2	Left	LET	33C2	–	Closed
6	58	M	3	Left	HET	33C3	–	Open, grade I
7	68	M	2	Right	LET	33C3	–	Closed
8	68	F	2	Left	LET	33A3 [VB1]	TKP	Closed
9	71	F	3	Right	HET	33A3	–	Closed
10	71	F	3	Left	LET	33A3 [VB1]	TKP	Closed
11	71	F	2	Left	-	33C2	–	Closed
12	71	F	3	Left	LET	33A3 [VB1]	TKP	Closed
13	71	F	3	Right	LET	33C2	–	Closed
14	76	F	3	Left	LET	33A3	–	Closed
15	81	F	3	Left	LET	33A3 [VB1]	TKP	Closed

*ASA* American Standardisation Association; *MOI *mechanism of injury; *AO/OTA* American Orthopaedic Trauma Association; *M *Male; *HET *high energy trauma; *F *Female; *LET *low energy trauma; *TKP *total knee prosthesis; * Gustilo and Anderson classification

Initial treatment strategies differed; eleven patients were treated with single lateral plating alone, four patients received plating combined with bone grafting (Table 2). Presumed underlying nonunion causes were lack of medial cortical support resulting in a persistent medial gap (14/15) and inadequate lag-screw fixation and a too short plate (1/15). In addition, preoperatively taken computed tomography (CT) scans were screened for sclerotic closure of the marrow canal at the proximal and distal femur fragment around the nonunion. The canal appeared open on both sides in six patients, and closed in seven. In one patient, only the proximal fragment appeared open. For one patient, there was no pre-operative CT scan available. No patients presented with an active infection (i.e. no draining sinuses or open wounds).

**Table 2:** Fracture history, nonunion treatment and outcomes

Patient	Initial DFF treatment	Number of revisions	Revision surgery type(s)	Duration of nonunion (mo)	W&Ç	NUSS	Nonunion treatment	Additional bone graft	Time to union (mo)	FU (mo)
1	AxSOS locking plate	2	VA distal femur LCP + ICBG + DBX (1, 2)	11	OT	44	VA distal femur LCP + medial PHILOS plate	ICBG	6	30
2	LISS plate	1	NCB plate + allograft bone chips	13	OT	34	95-degree condylar blade plate + medial PHILOS plate	ICBG + autologous hypertrophic callus	15	25
3	Lateral LCP	3	Bone grafting with 2 allograft femoral heads (1), medial distal femur plate + right ICBG + femoral head (2), removal of loose screws (3)	20	OT	46	95-degree condylar blade plate + medial PHILOS plate	ICBG + DBX	11	12
4	LISS plate	7	Incision + drainage (1, 2), Lateral VA distal femur LCP + anterior LCP + ICBG + DBX (3), incision + drainage (4), anterior plate removal + gentabeads (5), removal of gentabeads + antibiotic cement coated LISS plate (6), debridement + antibiotic cement coated LISS plate (7)	21	AT	58	VA distal femur LCP + medial PHILOS plate	ICBG	15	29
5	DCS	0	-	7	HT	26	LISS plate + medial PHILOS plate	ICBG + femoral head allograft + DBX	4	18
6	LISS plate	1	VA distal femur LCP + fibula allograft + DBX	12	OT	40	95-degree condylar blade plate + medial PHILOS plate	ICBG + DBX	9	25
7	LISS plate	1	95-degree condylar blade plate + ICBG	12	OT	34	95-degree condylar blade plate + medial PHILOS plate	ICBG + DBX	3	3
8	LISS plate	1	95-degree condylar blade plate + ICBG	17	OT	34	VA distal femur LCP + medial PHILOS plate	ICBG + DBX	6	51
9	Lateral LCP + calcium-phosphate	1	95-degree condylar blade plate + ICBG + DBX	23	OT	32	VA distal femur LCP + medial PHILOS plate	ICBG	54	54
10	VA distal femur LCP	0	–	13	HT	32	VA distal femur LCP + medial PHILOS plate	ICBG	3	4
11	Lateral LCP + homologous bone graft	5	Allograft croutons (1), ICBG (2), + quadriceps release (3), 95-degree condylar blade plate + ICBG (4), 95-degree condylar blade plate + ICBG + DBX (5)	41	HT	44	Medial PHILOS plate	ICBG	2	11
12	AxSOS locking plate	1	ICBG	11	OT	34	VA distal femur LCP + medial PHILOS plate	ICBG	15	56
13	AxSOS locking plate	0	-	7	OT	30	AxSOS locking plate + medial PHILOS plate	ICBG + femoral head allograft	3	3
14	LISS plate	3	LISS plate + cerclage around prosthesis (1), reposition LISS plate (2), croutons + ICBG (3)	20	OT	50	95-degree condylar blade plate + medial PHILOS plate	ICBG + DBX	8	25
15	NCB plate + allograft croutons + DBX	0	–	7	HT	28	VA distal femur LCP + medial PHILOS plate	ICBG + DBX	3	14

*DFF *distal femoral fracture; *mo *months; *W&Ç *Weber&Çech; *NUSS *Non Union Severity Score; *FU *Follow Up; *VA *variable angle; *LCP *locking compression plate; *ICBG *iliac crest bone graft; *DBX *demineralized bone matrix; *OT *oligotrophic; *PHILOS *proximal humeral internal locking system; *LISS* less invasive stabilization system; *NCB *non-contact bridging; *AT *atrophic; *DCS *dynamic condylar screw; *HT *hypertrophic

All patients had undergone multiple previous surgeries (median 2; range 2–8). Median time from initial DFF treatment to our index procedure was 13 months (range 6.8–40.5). Nonunions were classified as hypertrophic (*n* = 4), oligotrophic (*n* = 10) or atrophic (*n* = 1) according to the Weber&Çech classification system. Median nonunion severity score (NUSS) was 34 (range 26–58) (Table 2).

Seven patients received a VA-LCP, six a 95-degree condylar blade plate (in one patient the angular blade plate was not replaced), one a LISS plate and one an AxSOS locking plate. For medial fixation, all patients received the Philos plate, but different lengths were used: short in three patients, Philos long with 5 shaft holes for one patient, with 7 holes for one patient, with 8 holes for eight patients and with 10 holes for two patients. We err on the long size when choosing a plate size. All patients received autologous cancellous ICBG. In 12 patients, this was supplemented with a tricortical ICBG. In 7 patients, ICBG was insufficient so either 5 or 10 cc allograft demineralized bone matrix (DBM, DePuy Synthes) was added. In two patients, we added a femoral head allograft because the defect was larger than could be filled with bone chips (Table 2). Per-operatively taken cultures showed positive results in three patients (resp. staphylococcus epidermidis (*n* = 2) and staphylococcus hominis (*n* = 1). All three patients received antibiotics. One patient continued levofloxacin (oral) and rifampicin (oral) for 3 months. The second patient was given vancomycin intravenous via a peripherally inserted central catheter (PICC) line for two weeks followed by clindamycin (oral) and rifampicin (oral) for 2.5 months. For the patient who was infected with staphylococcus hominis, no specifications on antibiotic treatment were documented. All fifteen patients were discharged in good clinical condition after a median of eight days (range 4–111).

**Fig. 2:**
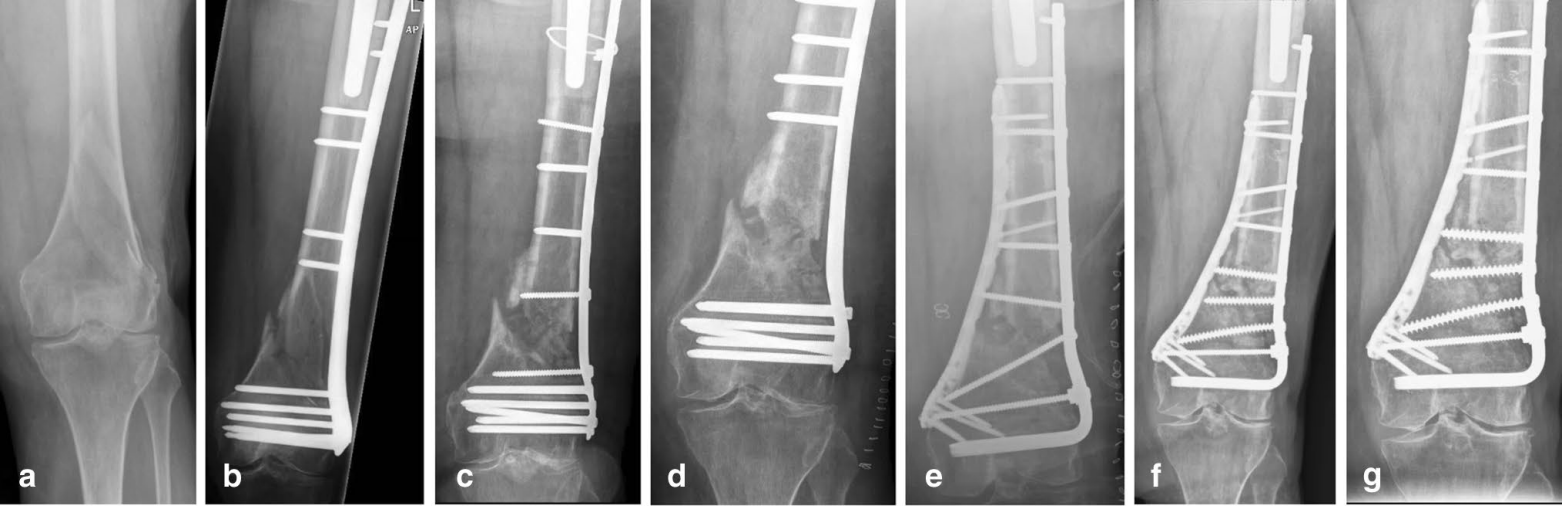
A seventy-six year old female was referred to us after several attempts to heal a comminuted extra-articular distal femur fracture (33A3) (**a**). Initial fracture treatment in another institution consisted of a LISS plate (**b**). Revision surgery with a new LISS plate and cerclage around hip prosthesis was performed 8 months after initial fracture treatment (**c**). Three days later a second revision surgery with a LISS plate was performed due to malposition of the previous plate (**d**). Six months later the patient underwent additional bone grafting surgery and was then referred to us with a persistent nonunion. At 20 months after the initial fracture we performed our index nonunion surgery with a 95-degree condylar blade plate and a Philos plate medially. In addition, autologous bone grafting was performed (**e**). Another bone grafting surgery with addition of three cancellous screws was performed six months later. Union was confirmed 2 months later (**f**). Radiographic imaging 19 months after index nonunion surgery show bone union and a well preserved knee joint (**g**)

Median follow-up was 24 months (range 3–56). Twelve patients (80%) united without complications after our index nonunion surgery. Median time to union for this group was 4.8 months (range 1.6–15). Three patients underwent additional bone grafting. Eventually all patients united (100% union) (Table 2, Fig. 2).

### Subsequent surgeries and complications

Three patients needed additional surgeries to procure healing. Two received surgeries at respectively 2 and 6.5 months after the index operation. One patient received additional posterior ICBG and united after 9 months. The second patient also received posterior ICBG with addition of three cancellous screws and united 2 months later. The third patient visited us when we contacted her as part of this study. Co-incidentally, she had noted to have progressive pain in the distal femur. A persistent nonunion was seen on CT. We decided on additional bone grafting surgery, performed 51 months after the index surgery. She united three months later.

One patient underwent a Judet quadricepsplasty for a stiff knee (arc of motion 30 degrees) one month after union was confirmed.

### Functional outcome

Ten patients completed the KOOS questionnaire at a median of 27 months (range 14–56). Five patients did not complete the questionnaire due to the following reasons: non-responders (*n* = 2), TKP (*n* = 2), death (*n* = 1). The median KOOS score of all subscales (5) combined was 76 out of 100. For pain respectively 86 (range 58–100), for other symptoms 77 (range 51–82), for activities of daily living 89 (range 51–99) and for quality of life 50 (range 19–63). Only two patients (aged 21 and 56) completed the questions on sport and recreative activities. Other patients did not participate in any sport or recreative activities because of their age. Overall patient satisfactory was good and the median range of knee motion (ROM) was 100 (range 70–130).

## Discussion

Nonunions of the distal femur are difficult to treat. Often, multiple revisions are needed. A single lateral plate may not be sufficient in providing the required stability. Lack of medial cortical support will likely result in a persistent medial gap, increasing the chance of varus collapse and hardware failure. Poor bone quality and stiffness further complicate revision surgeries on the distal femur.

We have used the Philos plate for distal femoral nonunions. Currently there is no specific plate designed for the medial distal femur. The Philos plate was developed for the proximal humerus. Interestingly, we –and others- use it in various other locations (e.g. proximal femur, wrist, ankle, proximal tibia). We especially like the titanium Philos for the distal femur because it shape fits nicely on the medial condyle and it has a small footprint that does not interfere with most knee prostheses. We first fix the plate distally with unicortical locking screws to not interfere with the lateral fixation. More proximally we use standard (non-locking) screws in the narrow part of the plate. Because of the flexibility of the plate, these screws contour the plate to the shaft providing a low profile. The most proximal screw can be placed percutaneously via a stab incision. Because of the trapezoid shape of the distal femur when viewed axially, the direction of the distal (locking) screws is from anteromedial to posterolateral providing stability in the coronal and sagittal plane. In the future, for this specific fracture/nonunion configuration, an anatomic medial distal femur plate (ideally with variable angle locking) may be helpful.

Using our technique with the Philos plate as medial buttress, we were able to achieve an initial union rate of 80%, eventually all patients (100%) united.

Medial stability can be obtained using various techniques. Wang and Weng [18] treated 13 patients using internal fixation combined with an allograft strut and autologous bone grafting. They report a 100% union rate at an average of 5 months and speculate that the added fixation with the allograft strut might be comparable with that of a dual plating technique. However, a relatively high proportion of their patients showed functional impairment. A study by Kanakeshwar et al. [9] treated 22 distal femur nonunions using locking plates in combination with an allograft strut and autologous bone grafting reports a 100% union rate at an average of 6.2 months. Their average age of 39 years is however lower than of our patients. Another study by Matelic et al., [19] obtained medial stability using endosteal substitution with a medial plate for recalcitrant nonunions of the femur. Seven patients, of which 3 patients with a DFN, were treated. Although the results indicate adequate union rates, two of three patients with a DFN needed additional surgeries. In a more recent analysis by Al Farii et al., [20] an almost similar technique with an endosteal plate was used, but this study concerned a very heterogenous group of patients with acute distal femur fractures instead of nonunions. Their results have also been questioned by Shekhar et al., [21] and they highlight the fact that additional medial cortical support is not routinely indicated for extraarticular metaphyseal fractures.

The application of medial distal femoral plates has been controversial because of the concern for damage to the branching arteries of the deep femoral artery (DFA) and the distal femur nerves. Cadaveric studies suggest however, that medial plate application can be performed safely [22, 23].

In a similar study, Holzman et al. [10] treated patients with the addition of a medial locking plate (large fragment 4.5 mm LCP). However, none of the patients received both plates in one surgical procedure, but only after (repeated) lateral plating. They report high union rates (19/20), but several complications did occur. We show similar union rates and fewer complications and suggest that both lateral plate replacement and medial plate application can be performed safely during one procedure.

In a study by Chapman et al. [24], thirteen patients with a nonunion of the supracondylar region were treated with dual plating and bone grafting. The study used an anterior approach for full exposure of the knee joint. Although all patients except one healed without complications, five patients needed additional surgeries.

Studies that are more recent describe techniques on the addition of a medial plate using minimal invasive techniques comparable with the technique we used to apply the Philos plate. Eleven patients studied by Swentik et al., [6] were treated with small plates percutaneously placed along the medial border. A union rate of 80% was reported. Beeres et al., [25] described five patients with a DFN. A helical locking plate was used for the medial side and four received additional bone grafting.

A recent analysis of 62 patients by Liu et al., [26] showed superior outcomes for dual plating compared to single lateral plating. The dual plating group showed significant higher union rates (93.8% vs 56.7%).

Others have reported on the use of a mega-prosthesis as a salvage for a distal femur nonunion [27] as well as for severely comminuted distal femur fractures [28]. Our orthopedic oncology service regularly uses these mega-prostheses for reconstruction after a distal femoral tumor resection. We strongly believe that all fractures and nonunions have tremendous intrinsic capacity to heal. Therefore, only in case of severe pre-existing degenerative, post-traumatic or rheumatoid arthritis of the knee we will consider a mega-prosthesis for a DFN. The risks of infection, residual instability and/or pain are simply too high. In addition, they are very expensive.

A study by Rajasekaran et al., [29] proposed an algorithm, including length of medial gap, for recalcitrant distal femur nonunions (RDFN). They were able to obtain an impressive success rate of 98%. Since almost all of our patients presented with a medial gap we tried to measure the length and volume of the gap using their pre-index CT scans. Given the retrospective design, it was impossible to accurately measure both factors. For a reliable measurement, we would suggest measuring the length of the gap intra-operatively after correction of alignment, as was done by Rajasekaran et al. [29]

Our study has –usual– limitations. The retrospective design resulted in difficulty obtaining comparable pre-operative clinical records. Second, all operations were performed by an orthopedic trauma surgeon with extensive experience in treating nonunions, making generalizability difficult. Finally, our patient population was quite heterogeneous and small.

Strengths of the study are that is it the first description of an intuitively attractive minimally invasive augmentation. No patient was lost to follow up. We used a patient-based outcome score as well as the NUSS to assess nonunion severity.

Obviously, as this is the first study using the Philos plate as a medial buttress for distal femoral nonunions, it is difficult to compare the results to other related reports. The technique described herein seems to be a safe and reproducible technique to address distal femur nonunions. Almost all our patients underwent multiple –unsuccessful- surgeries to treat their DFN, but did not unite until we performed our dual plating surgery with the Philos plate. For now, dual plating nonunions of the distal femur with a long lateral plate, bone grafting and a minimally invasive placed medial Philos buttress plate is our Gold standard.
